# Mathematical model for the relationship between single-cell and bulk gene expression to clarify the interpretation of bulk gene expression data

**DOI:** 10.1016/j.csbj.2022.08.062

**Published:** 2022-09-05

**Authors:** Daigo Okada, Cheng Zheng, Jian Hao Cheng

**Affiliations:** Center for Genomic Medicine, Graduate School of Medicine, Kyoto University, South Research Bldg. No.1(5F), 53 Shogoinkawahara-cho, Sakyo-ku, Kyoto 6068507, Kyoto, Japan

**Keywords:** Gene expression, Cellular heterogeneity, Single cell, Probability distribution, Differential expression analysis, Differential variability analysis

## Abstract

Background. Differential expression analysis is a standard approach in molecular biology. For example, genes whose expression levels differ between diseased and non-diseased samples are considered to be associated with that disease. On the other hand, differential variability analysis focuses on the differences of the variances of gene expression between sample groups. Although differential variability is also known to capture biological information, its interpretation remains unclear and controversial. Recent single-cell analyses have revealed that differences between sample groups can affect gene expression in a cellular subset-specific manner or by altering the proportion of a particular cellular subset. The aim of this study is to clarify the interpretation of mean and variance of bulk gene expression data.

Method. We developed a mathematical model in which the bulk gene expression value is proportional to the mean value of the single-cell gene expression profile. Based on this model, we performed theoretical, simulated and real single-cell RNA-seq data analyses.

Result and Conclusion. We identified how differences in single-cell gene expression profiles affect the differences in the mean and the variance of bulk gene expression. It is shown that differential expression analysis of bulk expression data can overlook significant changes in gene expression at the single-cell level. Further, differential variability analysis capture the complex feature affected by different gene expression shifts for each subset, changes in the proportions of cellular subsets, and variation in single-cell distribution parameters among samples.

## Highlights

• Mean and variance in bulk expression capture different features of gene expression.
• Differential expression analysis can miss significant changes in single-cell level.
• Change in proportions of cellular subsets can cause differential variability.
• Different expression shifts for cellular subsets can cause differential variability.

## Graphical abstract

**Figure:**
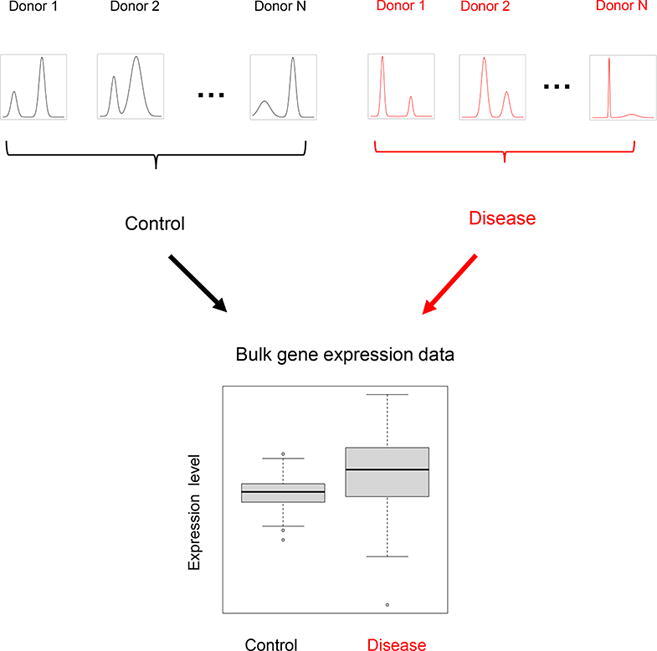

## 1 Introduction

**Fig. 1:**
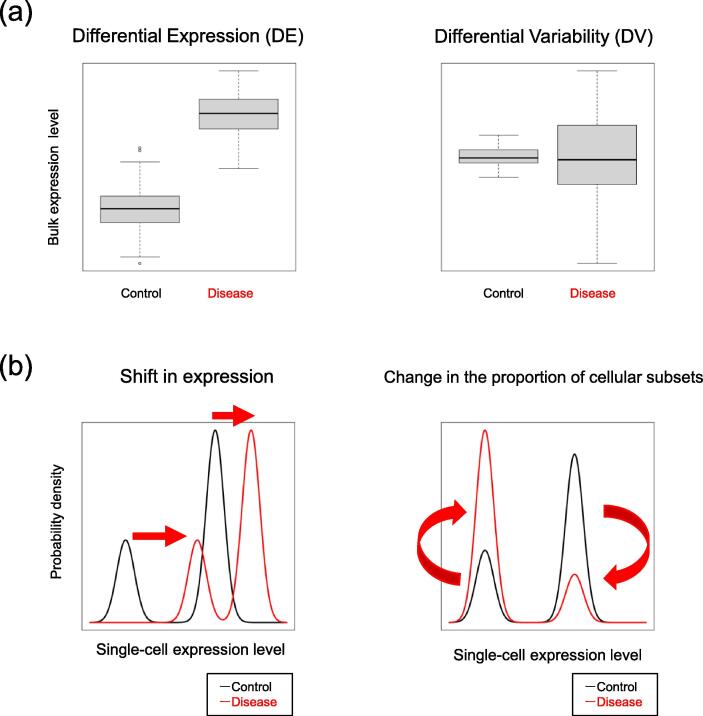
Background of the study. (a) Concept of differential expression (DE) and differential variability (DV) in gene expression analysis. DE and DV analysis capture the mean and the variance of bulk expression distributions between groups, such as control vs. disease groups, respectively. (b) Graphical illustrations showing changes in the distribution of two types of single-cell expression; the first type is characterized by a shift in expression levels in each cellular subset (left). The magnitude and direction of the shift can differ for each cellular subset, and can be expressed as a change in the individual distributions that make up the single-cell expression distribution. The second type is a change in the cellular subset proportion (right). This can be expressed as a change in the proportion of the component distribution.

Performing differential expression (DE) analysis of different sample groups is a standard approach in molecular biology. In recent years, transcriptome data have been used to comprehensively identify DE genes in different experimental groups [1], and several bioinformatics methods have been developed for this purpose [2], [3], [4]. In DE analysis, if gene expression levels differ significantly between diseased and non-diseased donors, then the genes are considered to be associated with that disease. Similarly, a comparison of gene expression data from different tissues or anatomical regions can therefore be used to identify tissue/region-specific genes [5], [6], [7]. Expression quantitative trait locus analysis (eQTL) can be used to identify genetic variants in genotype groups that are significantly associated with gene expression levels, and in so doing, can facilitate an understanding of the mechanisms underlying gene regulation and interpretations of functional genetic variants [8], [9]. Specifically, these DE analyses identify differences in mean expression values for bulk gene expression data between the groups based on disease, tissue or genotype (Fig.1(a)).

On the other hand, differential variability (DV) analysis is another approach for identifying differences in gene expression [10]. DV analysis captures differences in variance of gene expression values between the groups (Fig.1(a)). DV can capture biological information about a target disease or trait. To date, studies have employed DV analysis of transcriptome data to provide biological insights about disease and aging [11], [12], [13], [14]. For example, a strong relationship has been reported between variability in gene expression and a chronic lymphocytic leukemia subtype [14]. In the context of eQTL analysis, the genetic loci associated with variance in bulk gene expression value are discussed as expression variability QTL (evQTL) [15]. Although the biological processes underlying DV have been investigated from a biological standpoint, such as gene expression noise or epigenetics [16], the interpretation of DV of gene expression remains unclear and controversial.

Due to cellular heterogeneity, bulk gene expression data in DE or DV analyses are not typically sufficient for capturing the changes in the gene expression profiles of a cell population. Each sample in an experiment contains randomly selected cells, and each cell has a different gene expression level. Consequently, the cell population profile can be expressed as a probability distribution of gene expression, and the bulk gene expression data captures information of the average value of this distribution [17]. Recent advances in single-cell analysis have reported the existence of two different types of changes in a single-cell expression profile; shifts in gene expression and changes in the proportion of cellular subsets.

First, group differences shift the level of gene expression in the cell, and the direction and magnitude of this shift depends on the cellular subset. For example, it is known that tumor cell sub-populations show distinct drug responses [18]. The recent studies combining single nucleotide polymorphism (SNP) genotype data with single-cell RNA-seq (scRNA-seq) data or cytometry has shown that the effects of genetic variants on gene expression may differ depending on cellular subsets [19], [20], [21]. Such changes in the single-cell expression distribution can be expressed as shown in the left panel of Fig.1(b).

Second, group differences change the proportion of cellular subsets, as shown in the right panel of Fig.1(b). Differences among groups can alter proportion of cellular subsets by affecting cell differentiation, maturation and transformation. Studies have been conducted to identify cellular subsets with different proportions between sample groups [22], [23]. Previous studies combining SNP genotype and cytometry analyses identified SNPs associated with different lymphocyte subsets [24], [25]. In addition, it has been suggested that a large number of SNPs are associated with individual differences in lymphocyte profiles, even though their effects are small [25]. Changes in the proportion of cellular subsets can affect the bulk gene expression value. For example, when the proportion of a cellular subset with a relatively high gene expression level increases, the bulk expression levels also increase.

In the complex physiological and pathological changes that occur within cells, both shifts in gene expression and changes in cellular subset proportions can occur simultaneously, resulting in a combined contribution to the bulk expression value. For example, both of them in intestinal epithelial cell population have been observed in patients with Crohn’s disease [26]. Evaluating how these changes in single-cell expression profiles are manifested in DE and DV genes is central to understanding the biological mechanisms underlying both DE and DV analysis. Further, given the increased interest in single-cell expression analysis in recent years, it is important to evaluate the results of single-cell expression analyses and compare them to the results of bulk expression analyses that have been reported to date.

In this study, we describe a mathematical model for examining bulk gene expression levels and the single-cell expression distribution behind them. Specifically, single-cell expression profiles are modeled using a mixed probability distribution, and the relationships among their parameters and the mean and variance of the bulk expression values among samples are clarified. The model proposed in this study clarifies the interpretation of DE and DV analysis and provides new insights into the relationship between bulk and single-cell data analyses.

## 2 Methods

### 2.1 Mathematical model for relationship between a single-cell expression profile and a bulk gene expression value

**Fig. 2:**
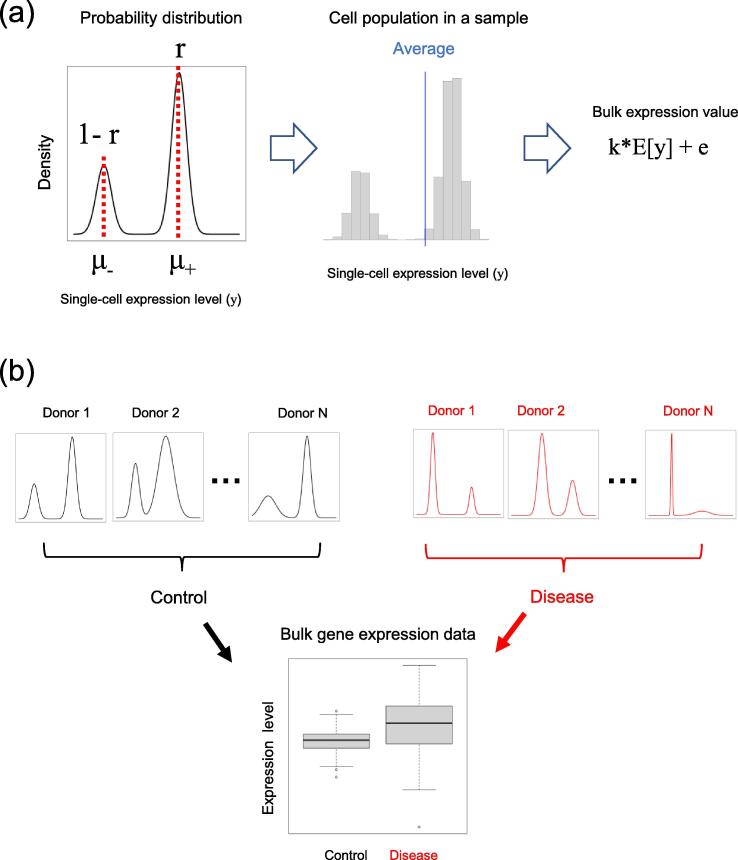
Mathematical model of the relationship between single-cell expression profiles and bulk expression data. (a) Model of the relationship between the single-cell expression profile and bulk gene expression values. The single-cell expression profile is modeled as a mixture distribution. $\mu_{+}$ and $\mu_{-}$ are the expected values of the component distributions and *r* is the proportion of cellular subset. Bulk samples are obtained as statistical samples where the number of cells contained is sufficiently large. Bulk gene expression values are proportional to the expected value of the population distribution  + the measurement error (k*E[y]+e). (b) Model of the bulk expression data analysis. In the bulk data analysis, multiple sample values are taken where $\mu_{+},\mu_{-}$, and *r* have different values in each sample. This mathematical model evaluates the relationships among the statistics of $\mu_{+},\mu_{-}$ and *r*, and the bulk gene expression *Y*.

We developed the following mathematical model to clarify the relationship between one single-cell expression profile and its bulk gene expression value for one gene (Fig.2(a)). The bulk samples of multicellular organisms consist of many cells, which show cellular heterogeneity and consist of multiple cellular subsets. The distribution of gene expression for these cells can be expressed as a mixture distribution of those of different cellular subsets.

To evaluate the effect of a specific cellular subset, we used cellular subset + and cellular subset-. The single-cell expression value for a cell of cellular subset + is assumed to follow a probability distribution $f_{+}(y)$ with mean $\mu_{+}$. Similarly, that for a cell in cellular subset- is assumed to follow a probability distribution $f_{-}(y)$ with mean $\mu_{-}$. Let the proportion of cellular subset + be *r* and that of cellular subset- be 1-r. Due to genetic and environmental effects, the parameters affecting the single-cell expression distributions are assumed to vary among donors. We modeled $\mu_{+},\mu_{-},r$ by assuming that these are random variables that are independent of each other.

Under this model, the single-cell expression level *y* follows a mixture probability distribution, as follows:

(1) $$y∼{\mathrm{rf}}_{+}(y)+(1-r)f_{-}(y)$$

The bulk gene expression value (*Y*) can be considered as the mean value for a single-cell expression distribution. If the bulk sample contains a sufficient number of cells, then the bulk gene expression level (*Y*) can be modeled with a proportionality constant *k* and measurement error (*e*) as follows:

(2) $$\begin{array}{cc} Y= & \mathrm{kE}[y]+e\\ = & \mathrm{kr}\mu_{+}+k(1-r)\mu_{-}+e \end{array}$$

where, *e* is assumed to be independent of other random variables $\mu_{+},\mu_{-},r$.

For simplicity, we set k = 1 in the discussion.

(3) $$Y=r\mu_{+}+(1-r)\mu_{-}+e$$

As a result, the group difference in bulk expression data, such as control vs. disease, are interpreted via the single-cell gene expression distribution according to Eq. 3. In DE analysis, the bulk data for the disease group (Group D) and the control group (Group C), $E[Y^{D}]-E[Y^{C}]$ can be detected statistically as the difference of bulk expression values for each group. This model allows us to mathematically evaluate the relationship between the parameters for the single-cell expression distribution with cellular heterogeneity and the results of the bulk gene expression analysis (Fig.2(b)).

### 2.2 Relationships among cellular heterogeneity, DE and DV analyses in the bulk experiment

From Eq. 3, the bulk expression value *Y* can be written as

(4) $$E[Y]=E[r]E[\mu_{+}]+(1-E[r])E[\mu_{-}]$$

Consider the case of comparing bulk data for disease group (Group D) and the control group (Group C). In Group D, the expected value for the parameters of single-cell expression distribution in the model is shifted from those of group C, as follows:

(5) $$\begin{array}{cc} & E[\mu_{+}^{D}]=E[\mu_{+}^{C}]+\Delta \mu_{+}\\ & E[\mu_{-}^{D}]=E[\mu_{-}^{C}]+\Delta \mu_{-}\\ & E[r^{D}]=\alpha_{r}E[r^{C}] \end{array}$$

where $\Delta \mu_{+},\Delta \mu_{-}$ and $\alpha_{r}$ express the difference in $\mu_{+},\mu_{-}$ and *r* between groups. Note that $\alpha_{r}E[r^{C}]$ is restricted to the range 0 to 1. The differential expression between the two groups, i.e., $E[Y^{D}]$ - $E[Y^{C}]$, can be expressed as follows, based on Eq. 4 (see Appendix A for details).

(6) $$E[Y^{D}]-E[Y^{C}]=(E_{+}^{C}-E_{-}^{C})(E_{r}^{C}(\alpha_{r}-1)+\alpha_{r}E_{r}^{C}d_{+}+(1-\alpha_{r}E_{r}^{C})d_{-})$$

where, $d_{+}=\frac{\Delta \mu_{+}}{\left(E_{+}-E_{-}\right)}$ and $d_{-}=\frac{\Delta \mu_{-}}{\left(E_{+}-E_{-}\right)}$. In addition, we let $E_{+}^{C},E_{-}^{C}$ and $E_{r}^{C}$ equivalent to $E[\mu_{+}],E[\mu_{-}]$ and E[r] in Group C, respectively.

Depending on the combination of $\alpha_{r},d_{+}$, and $d_{-},E[Y^{D}]-E[Y^{C}]$ can take a value of zero and never be identified by DE analysis, even though positive activation of gene expression is occurring at the single-cell level. Based on these results, if a group difference affects both the cellular subset proportion and the gene expression level in each cell, then it can be missed in the bulk gene expression analysis.

Indeed, if $d_{+}>0$ and $d_{-}>0$, then the condition for $E[Y^{D}]-E[Y^{C}]\leq 0$ can be expressed as follows (from Eq. 6):

(7) $$\alpha_{r}\leq \frac{1-\frac{d_{-}}{E_{r}^{C}}}{1+d_{+}-d_{-}}$$

where we assumed $E_{+}^{C}>E_{-}^{C},r>0,1+d_{+}-d_{-}>0$. $E[Y^{D}]=E[Y^{C}]$ is satisfied when the equal sign holds.

On the other hand, variance in the bulk gene expression value can be calculated from Eq. 3, as follows:

(8) $$V[Y]=V[r\mu_{+}+(1-r)\mu_{-}+e]$$

From Eq. 8, the following relationship can be mathematically derived (see Appendix B for details):

(9) $$V[Y]={\left(E_{+}-E_{-}\right)}^{2}V_{r}+E_{r}^{2}V_{+}+{\left(1-E_{r}\right)}^{2}V_{-}+(V_{+}+V_{-})V_{r}+V[e]$$

In addition, we let $E_{+},E_{-},E_{r},V_{+},V_{-}$ and $V_{r}$ be equivalent to $E[\mu_{+}],E[\mu_{-}],E[r],V[\mu_{+}],V[\mu_{-}]$ and V[r], respectively. Eq. 9 suggests that there are three main factors that explain the differential variability between groups. First, $V_{+},V_{-}$ and $V_{r}$ directly affect the variability in bulk gene expression. Second, the change in $E_{r}$ affects the variability in bulk gene expression via the term $E_{r}^{2}V_{+}+{\left(1-E_{r}\right)}^{2}V_{-}$. If the group difference increases the more variable subset proportion, then V[Y] can be increased. Third, ${\left(E_{+}-E_{-}\right)}^{2}$ can affect V[Y]. If the group difference changes ${\left(E_{+}-E_{-}\right)}^{2}$, then it affects the bulk expression variance via the term ${\left(E_{+}-E_{-}\right)}^{2}V_{r}$. Importantly, even if $V_{-},V_{+}$ and $V_{r}$ do not change, a change in $E_{+},E_{-}$ and $E_{r}$ can change the variance in the bulk gene expression. Note that cell-to-cell variability V[y] does not appear in this equation.

### 2.3 Visualization of the difference between DE and DV genes

Based on the above theory, we visualized the relationship between the parameter ($d+,d-,\alpha_{r}$) and an increase or decrease in the mean and variance of the bulk gene expression. We focused on a situation where expression is increased in both cellular subsets ($d_{+}\geq 0,d_{-}\geq 0$) and the proportion of cellular subset + is decreased ($\alpha_{r}\leq 1$). For the combinations of equally spaced d + and d- (0, 0.1, 0.2, …, 1) and three patterns of $\alpha_{r}$ (0.2, 0.5, 0.8), we calculated the difference in the means and variances, and visualized the parameter space with an increase and a decrease in the mean and the variance of the bulk gene expression. We set other parameters in the model as follows: $E_{+}=2,E_{-}=1,E_{r}=0.5,V_{+}=0.3,V_{-}=0.1$ and $V_{r}=0.05$.

### 2.4 Simulation analysis of DV genes

We created a computational simulation scheme for the proposed mathematical model. First, this model contains fourteen parameters: *N* is the number of samples, $E_{+},V_{+},E_{-},V_{-},E_{r},V_{r},d_{+},d_{-}$ and $\alpha_{r}$ are the parameters that define individual differences in the distribution parameters described in the above model, *n* is the number of cells in the bulk sample, $V_{\mathrm{err}}$ is the measurement noise associated with quantifying the bulk expression value, and $V_{y+}$ and $V_{y-}$ are the variances of single-cell expression levels in each subset.

First, for *N* samples in the Group C, $\mu_{+},\mu_{-}$ and *r* are sampled from the Normal or uniform distributions as shown below, and assigned to each sample. Normal and uniform distributions are uniquely determined by the mean and variance parameters.

$$\begin{array}{cc} & \mu_{+}∼\mathrm{Normal}(E_{+},V_{+})\\ & \mu_{-}∼\mathrm{Normal}(E_{-},V_{-})\\ & r∼\mathrm{Uniform}(E_{r},V_{r}) \end{array}$$

Also, for *N* samples in the Group D, $\mu_{+},\mu_{-},r$ are sampled according to $d_{+},d_{-}$ and $\alpha_{r}$, based on Eq. 5.

$$\begin{array}{cc} & \mu_{+}∼\mathrm{Normal}(E_{+}+d_{+},V_{+})\\ & \mu_{-}∼\mathrm{Normal}(E_{-}+d_{-},V_{-})\\ & r∼\mathrm{Uniform}(\alpha_{r}E_{r},V_{r}) \end{array}$$

Next, we generated the single-cell expression value (*y*) of each sample based on Eq. 1, where $f_{+}(y)$ and $f_{-}(y)$ are modeled as normal distributions. From the following formula, we sampled *n* cells independently.

$$y∼\mathrm{rNormal}(\mu_{+},V_{y+})+(1-r)\mathrm{Normal}(\mu_{-},V_{y-})$$

After sampling *n* single-cell expression values, the bulk expression value is calculated as the mean of $y=[y_{1},y_{2}\ldots y_{n}]$ and a measurement error (*e*) is added.

$$\begin{array}{cc} & Y=\overset{¯}{y}+e\\ & e∼\mathrm{Normal}(0,V_{\mathrm{err}}) \end{array}$$

As a result, the bulk expression values for *N* control samples and *N* disease samples were simulated.

Based on this simulation scheme, we simulated two situations in which only the variance of the bulk expression is changed without changing the mean value. Eq. 9 shows that the difference in $E_{r}$ (Example 1) or ${\left(E_{+}-E_{-}\right)}^{2}$ (Example 2) can change the bulk expression variance, even if $V_{+},V_{-}$ and $V_{r}$ are the same in the two groups.

(Example 1) We simulated an example where the variance in bulk gene expression decreases as $E_{r}$ decreases in the disease group when $V_{+}>V_{-}$. The model parameters were as follows: $N=1000,E_{+}=2,E_{-}=1,E_{r}=0.9,V_{+}=1,V_{-}=0.1,V_{r}=0.001,n=10000,V_{\mathrm{err}}=0.1,V_{y+}=1$ and $V_{y-}=1$. $d_{+},d_{-}=0.5$ were used so that the value of $E_{+}-E_{-}$ was the same in the control and disease groups. $\alpha_{r}$ was set so that $E[Y^{C}]=E[Y^{D}]$was satisfied. We checked the simulated bulk expression data for the two groups to confirm whether the changes in the proportions of cellular subsets could cause DV.

(Example 2) We simulated an example where the variance in the bulk gene expression increases as $E_{+}-E_{-}$ increases. The model parameters were as follows: $N=1000,E_{+}=2,E_{-}=1,E_{r}=0.9,V_{+}=0.01,V_{-}=0.01,V_{r}=0.001,n=10000,V_{\mathrm{err}}=0.1,V_{y+}=1$ and $V_{y-}=1$. $V_{+}$ and $V_{-}$ were the same to repress the effect of differences in the proportions of different cells. Instead, $d_{+}=10$ and $d_{-}=0.1$ were used so that $E_{+}-E_{-}$ increases. $\alpha_{r}$ was determined so that $E[Y^{C}]=E[Y^{D}]$was satisfied. We checked the simulated bulk expression data for the two groups to confirm whether the different gene expression shifts for each subset could could cause DV.

### 2.5 Real single-cell RNA-seq data analysis

Here, we described the analysis of real single-cell RNA-seq data by applying our method to elucidate single-cell expression changes underlying bulk DE and DV genes. We used the public scRNA-seq dataset for ulcerative colitis (UC) (NCBI GEO ID:GSE125527) [27]. In this study, we used data for the processed scRNA-seq of human peripheral blood mononuclear cells (PBMCs) from seven patients with ulcerative colitis (UC) and eight control donors (NCBI GEO ID:GSM3576411-GSM3576425). After log(1  + count value) transformation, the sum of the gene expression values for each cell was normalized to be $10^{6}$ and used for downstream analysis.

The DE genes and DV genes were identified at the bulk level using the following procedure. Bulk gene expression levels for each sample were defined as the average of the single-cell expression values among cells. For the genes with an average bulk gene expression level of >20 in the 15 samples, we performed Welch’s t-test and F-test analyses to compare the UC and the control groups and calculated p values. We applied Benjamini-Hochberg (BH) correction [28] to the t-test p values and identified the genes with adjusted p values <0.05 as DE genes. We also applied BH correction to the F-test p values and identified the genes with adjusted p values <0.05 as DV genes.

For the identified top DE gene and top DV gene with the smallest p value, we estimated the parameters of single-cell expression distribution ($\mu_{+},\mu_{-}$ and *r*). In many cases, the single-cell expression distribution consists of the cell population with zero or small expression values, and cell populations with higher expression values. For the single-cell expression distribution of each gene in each sample, we applied kmeans clustering and divided the cells into subset + and subset-. By calculating the mean expressions and proportions of subset + and subset- cells, we could then estimate $\mu_{+},\mu_{-}$ and *r* for each sample. We then evaluated the distributions of these estimated parameters for the samples and identified differences in the single-cell expression distributions that underlie the bulk expression of DE and DV genes.

## 3 Results

### 3.1 Theoretical and simulation analyses

**Fig. 3:**
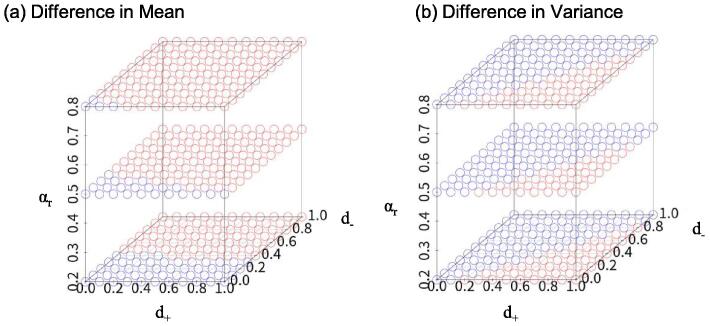
Example of theoretical analysis involving the comparison of the bulk mean and variance between two groups. (a) Example of plotting difference in mean expression for each combination of $d_{+},d_{-}$ and $\alpha_{r}$. Combinations where $E[Y^{D}]>E[Y^{C}]$ are plotted in red and combinations where $E[Y^{D}]\leq E[Y^{C}]$ are plotted in blue. Even if $d_{+}>0,d_{-}>0$, it is possible that $E[Y^{D}]\leq E[Y^{C}]$. (b) Example of plotting difference in variance for each combination of $d_{+},d_{-}$ and $\alpha_{r}$. Combinations where $V[Y^{D}]>V[Y^{C}]$ are plotted in red and combinations where $V[Y^{D}]\leq V[Y^{C}]$ are plotted in blue. DE and DV analysis capture different features of the single-cell distribution.

Fig. 3(a) shows $E[Y^{D}]-E[Y^{C}]$ for the combination of $d_{+},d_{-}$ and $\alpha_{r}$. Even though the expression shift is positive for subsets ($d_{+}\geq 0,d_{-}\geq 0$), the difference in mean expression value can be negative depending on $\alpha_{r}$. Fig.3(b) shows the difference in variance for the combination of $d_{+},d_{-}$, and $\alpha_{r}$. Compared to Fig.3(a), the plot pattern is different. These results indicate that DE and DV analysis capture different features of the single-cell distribution.

**Fig. 4:**
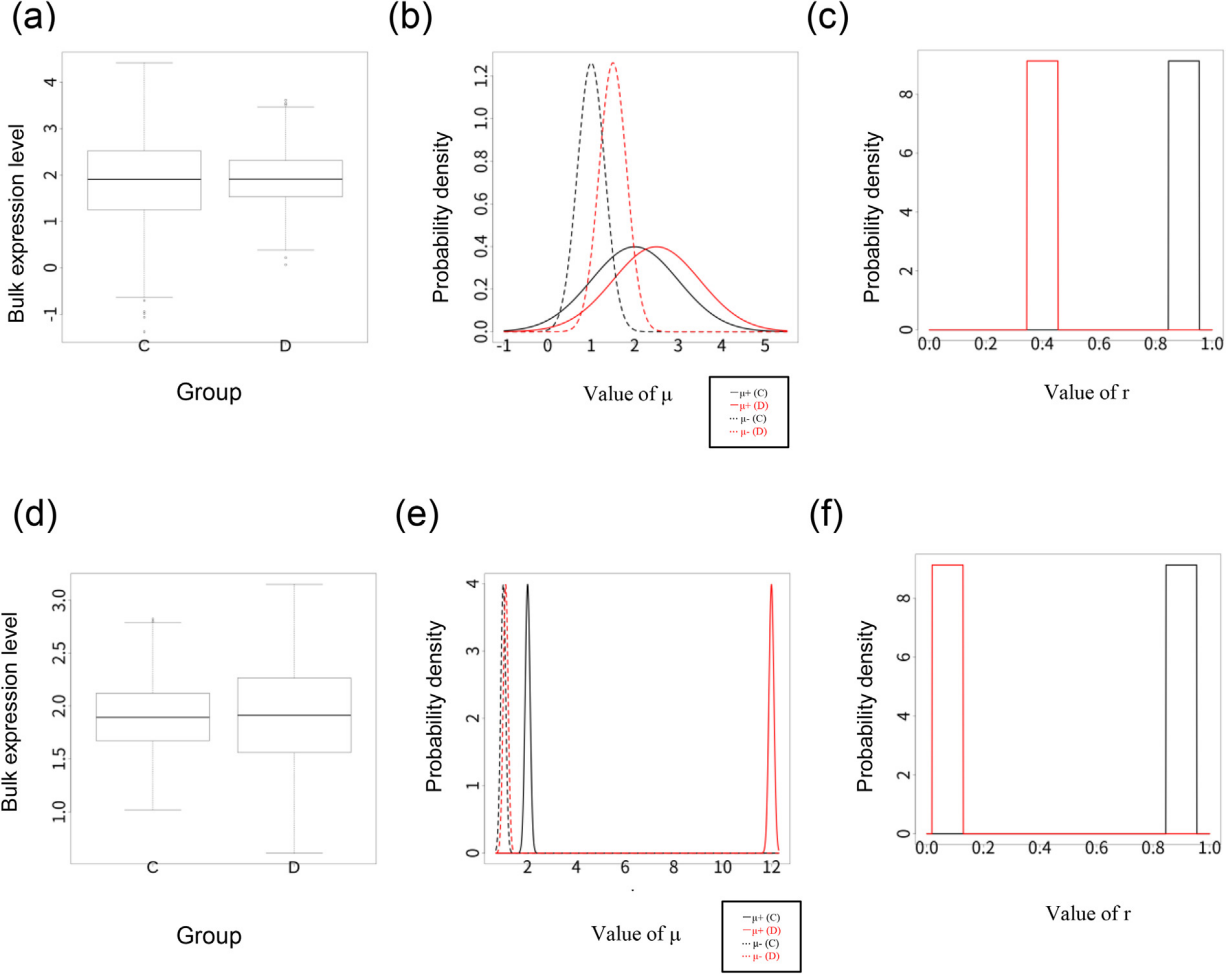
Results of the simulation analysis. (a) Bulk expression result with no mean difference and a significant difference in variance in Example 1 of simulation analysis. (b) and (c) are the distributions of $\mu_{+},\mu_{-}$ and *r* among samples in this simulation. (d-f) are the those of Example 2.

We simulated situations where the variance in bulk expression differed between sample groups without changing mean values. Fig.4(a) shows an example where only changes in the proportions of cellular subsets alters variance of bulk gene expression. This is because the subset + cells with large variance decrease in number and the subset- cells with small variance increase in number. Fig. 4, (b,c) shows the parameter distributions for $\mu_{+},\mu_{-}$ and *r* in the two groups in this simulation. Fig.4(d-f) shows another example where a shift in gene expression changes variance of bulk gene expression. These results show that a changes in the proportions of cellular subsets or a different expression shifts for cellular subsets can cause DV.

### 3.2 Real single-cell RNA-seq data analysis

We used a public human scRNA-seq dataset compiled using data for seven patients with UC and eight control subjects. The results of a bulk level analysis showed that there were 289 DE genes and four DV genes. No matches were observed between these DE and DV genes. We investigated the top DE and DV genes with smallest p value in detail.

**Fig. 5:**
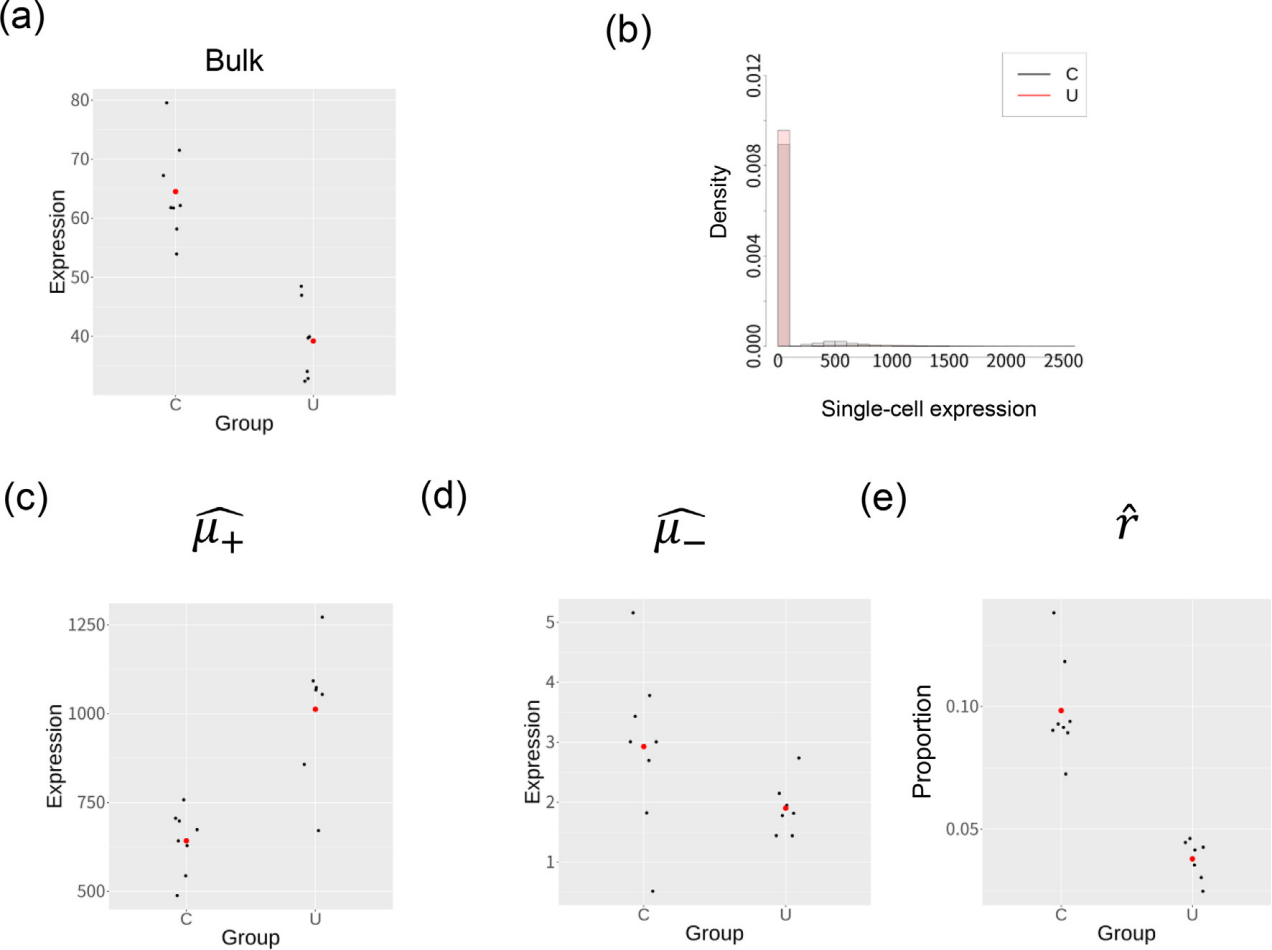
Analysis of top DE gene with the smallest p value (*POM121*). (a) Bulk gene expression levels of control (C) and ulcerative colitis (U) groups. The red points in the plot represent the mean of the bulk expression values for each group. (b) A histogram showing single-cell expression levels for the C and U groups, plotted for the pooled cells in all samples in each group. (c, d, e) Estimated parameters for single-cell expression distributions (μ+^,μ-^ and r^) for the samples. Red points represent the mean values obtained for each group.

The top DE gene in the bulk expression analysis was *POM121*, which had a mean bulk expression value that decreased significantly in UC group (Fig.5(a); t-test p value is $1.51\;*\;10^{-5}$). Fig.5(b) shows a histogram of single-cell expression values for pooled cells of each group, which shows that the expression level for this gene was low in a large number of cells (cellular subset -) and high in a small number of cells (cellular subset+). Fig.5, (c,d,e) shows the estimated $\mu_{+}$ and $\mu_{-},r$ values for each sample group. The findings suggest that the gene expression level increased markedly in subset + cells in UC group, while the proportion of these cells decreased. Although these two effects act in opposite directions, the greater influence of the former effect increases bulk gene expression.

**Fig. 6:**
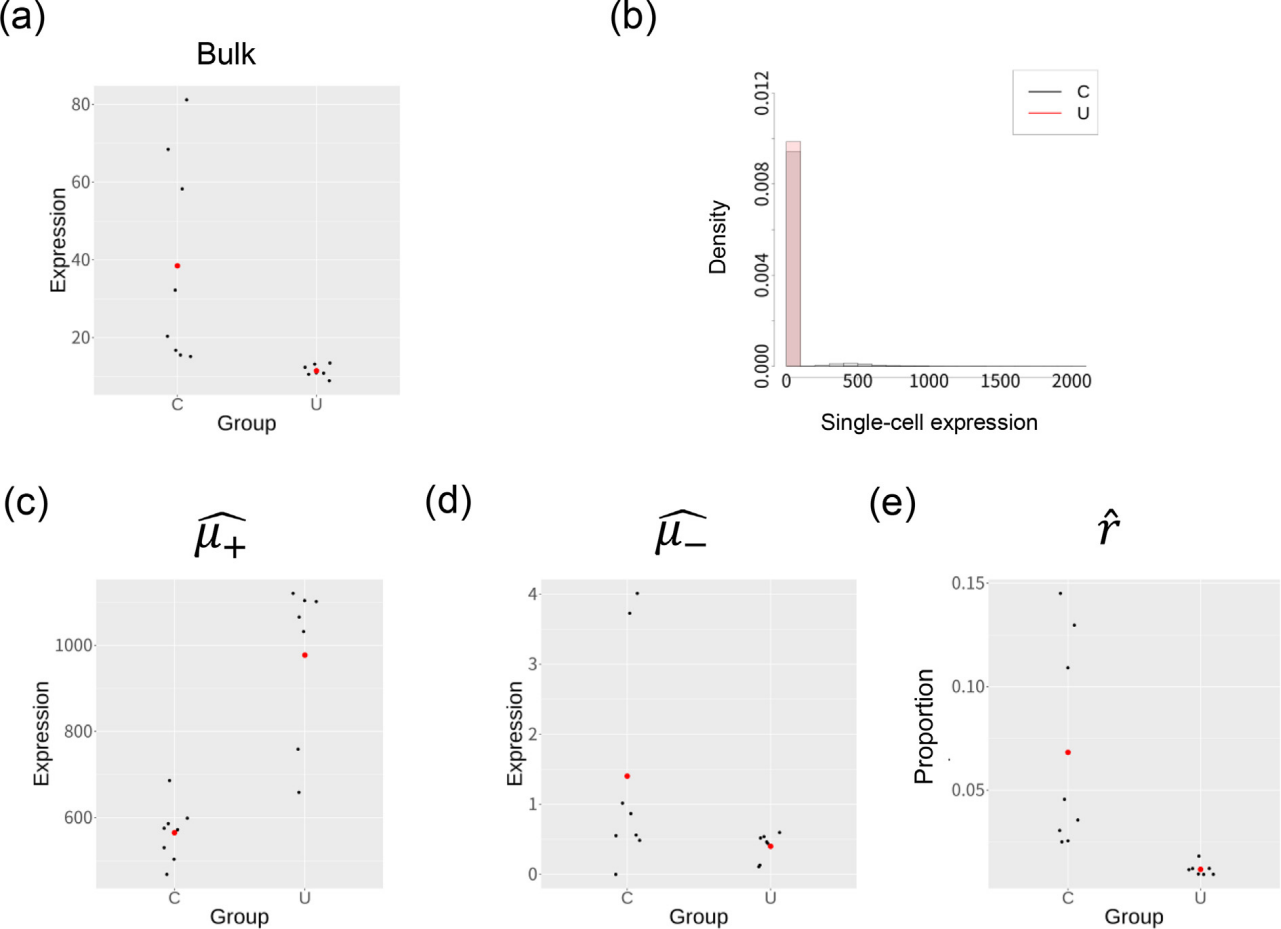
Analysis of the top DV gene with the smallest p value (*MAP1LC3B2*). (a) The bulk gene expression level of control (C) and ulcerative colitis (U) groups. The red points in the plot represent the mean of the bulk expression values for each group. (b) A histogram showing single-cell expression levels for C and U groups, plotted for the pooled cells in all samples for each group. (c, d, e) Estimated parameters of single-cell expression distributions (μ+^,μ-^ and r^) for the samples. Red points represent the mean values obtained for each group.

The top DV gene is *MAP1LC3B2* whose bulk expression variance is significantly decreased in UC group (Fig.6(a); the F-test p value is $8.77\;*\;10^{-7}$). Fig.6(b) shows a histogram of single-cell expression values for pooled cells of each group, which also shows that the expression level for this gene was low in a large number of cells (cellular subset -) and high in a small number of cells (cellular subset+). Fig.6, (c,d,e) shows the estimated $\mu_{+}$ and $\mu_{-},r$ values for each sample group. While the mean and variance of the bulk expression level decreased in UC group, $\mu_{+}$ increased. The large decrease in the mean and variance of *r* in UC group is considered to lead significant difference of variance.

## 4 Discussion

The proposed model provides the insights for the interpretations of DE and DV genes identified using bulk data. First, when changes in the proportions and expression shifts in cellular subsets occur, they may cancel each other out and not be detected in bulk data analysis. Second, underlying DV gene, there is a combined contribution of different expression shifts for cellular subsets, changes in the proportion of cellular subsets, and changes in individual differences in the parameters for single-cell expression profiles. These are important considerations when interpreting the results obtained from bulk expression analysis. Third, DV and DE capture different aspects of single-cell expression profile differences. In recent years, methods for directly detecting dissimilarities in single-cell expression distributions using scRNA-seq or cytometry data have been proposed [29], [30], [31], [32], [33]. Our findings provide an insight into the theoretical relationships among DE and DV gene identified in bulk experiment, and differential distributed genes identified in single-cell experiment.

Here we examine the difference between variability in the bulk expression data (V[Y]) and the cell-to cell variability (V[y]). Recently, studies on single-cell analysis have examined cell-to-cell variability and the statistical methods for analyzing this expression characteristics [34], [35]. Our analysis showed that the formula for V[Y] does not include V[y] and that there is no direct relationship between them when the sample contains a sufficiently large number of cells. Nevertheless, shifts in gene expression or changes in the proportion of cellular subsets will also induce changes in V[y] in the same direction in individual samples. In addition, changes in $V[y],V[\mu_{+}],V[\mu_{-}]$ and V[r] due to disease or aging may indicate the existence of a common mechanism for disruption of the control mechanism for biological phenomena.

The scRNA-seq expression data is characterized by the inclusion of many zero values [36]. Then, statistical models that can handle zero-inflation are often used in single cell data analysis. Specifically, it is often modeled by probability distributions such as negative binomial, poisson, zero-inflated negative binomial or zero-inflated poisson [36]. Our model is applicable to any probability distribution including the zero-inflation model. Since the relationship between expectation value of distribution and its parameters is known mathematically for these theoretical distributions, it is possible to calculate the values such as $E_{+}$ or $V_{+}$ from the mean and variance of the parameter values among samples, which provide the insights about the mean and variance of the bulk gene expression values. If future large scale single cell genomics studies will provide insight into the distribution of the parameters among samples, it will enhance our understanding of the relationship between single cell and bulk data analysis even more.

The analysis of real scRNA-seq data presented in this study is an effective tool for examining the relationship between scRNA-seq data and bulk data, but the study has several limitations. A major limitation is that the estimates obtained for E^+,E^-,E^r,V^+,V^- and V^r are not always accurate. These estimates can be affected by biases associated with clustering and parameter estimation. Also, the number of cellular subsets that are actually present in a tissue is not always known, and the identification and classification of cellular subsets using bioinformatic methods is a major research task in the field of single-cell genomics. Combining the mathematical model proposed in this study with advanced bioinformatics methods may further the study of single-cell genomics.

In our theoretical framework, the assumption that the bulk gene expression value is proportional to the mean value of the single-cell gene expression profile is essential. In real data analysis, the attention should be paid to whether this assumption holds. If very rare subset has non-zero expression value, the analysis will be susceptible to sampling bias. In such cases, it would be necessary to obtain data from a larger number of cells to capture the information of distribution. In addition, this assumption is also an unstable for low expressed gene because gene expression quantification by RNA-seq is unstable technically. Therefore, filtering the low expressed genes are important step as preprocessing under our theoretical framework.

The model described in this study could potentially be used in theoretical fields. Extending the model to poly-genes will allow more bulk expression analysis methods to be applied at the single-cell level. For example, gene co-expression network analysis is performed extensively in transcriptome analysis where it is used to infer biological processes and the roles of important transcription factor genes in complex traits [37], [38]. It is necessary to consider at least two genes when using the model to investigate correlations between gene expression. While gene co-expression analysis has been used to clarify relationships in gene regulation, it is unclear what exactly the identified relationships captures. Another extension would be a model that considers spatial information. When acquiring bulk gene expression data, not only information on the shape of the distribution but also spatial information is lost. In recent years, with the development of spatial genomics technology, single cell transcriptome data can be obtained with spatial information [39]. Since our framework works as a model of cellular population heterogeneity in general, it is possible to interpret cellular subset as spatial information. For example, it can also be used as a model for spatial information by setting $f_{+}(y)$ as the distribution on the specific region and $f_{-}(y)$ as those on another region. As candidate for future improvements, mathematical models that simultaneously consider cellular subsets and regional information can be considered.

On the application side, our mathematical model could be applied, not only to the analysis of gene expression data, but also to the analysis of arbitrary biomolecular expression data. For example, in the epigenome layer, an increase in the variability of DNA methylation intensity at the bulk level has been reported to be associated with aging [40], [41], [42], [43]. In recent years, single-cell expression data have been obtained for various omics layers. It is considered that the concepts presented in this study will be useful for reinterpreting molecular biology knowledge obtained at the bulk level using single-cell data.

## 5 Conclusion

In this study, we present a mathematical model to clarify single-cell expression profiles with cellular heterogeneity and bulk gene expression data. The model considered the shift in gene expression and changes in the proportion of cellular subsets. Theoretical and simulation analyses showed that the DE analysis can overlook significant changes in gene expression at the single-cell level. In addition, it is revealed that DV analysis capture the feature affected by different expression shifts for cellular subsets, changes in the proportions of cells, and variations in single-cell distribution parameters among samples. The model presented in this study effectively clarifies the differences in interpretation of DE gene and DV gene identified in bulk experiment and provides new insights into the relationship between bulk data analysis and single-cell data analysis.

## 6 Funding

This work was funded by a KAKENHI Grant-in-Aid from the Japan Society for the Promotion of Science (JSPS; Grant No. 21K21316).

## Code availability

The code used in this study is available athttps://github.com/DaigoOkada/ScBulkModel.

## Declaration of Competing Interest

The authors declare that they have no known competing financial interests or personal relationships that could have appeared to influence the work reported in this paper.

## Appendix A The detail of Eq. (6)

We calculated $E[Y^{D}]-E[Y^{C}]$ using the parameters for the single-cell expression distribution $\mu_{+},\mu_{-}$ and *r*. We use the following properties for the expected value of the sum and product of two random variables, A and B.

(A.1) E[A+B]=E[A]+E[B]

Further, if A and B are independent,

(A.2) E[AB]=E[A]E[B]

With the above formula, $E[Y^{D}]-E[Y^{C}]$ can be expressed as follows. Here, we let $E_{+}^{C},E_{-}^{C}$ and $E_{r}^{C}$ be equivalent to $E[\mu_{+}],E[\mu_{-}]$ and E[r] in control group, respectively.

$$\begin{array}{cc} & E[Y^{D}]-E[Y^{C}]\\ = & \alpha_{r}E_{r}^{C}(E_{+}^{C}+\Delta \mu_{+})+(1-\alpha_{r}E_{r}^{C})(E_{-}^{C}+\Delta \mu_{-})-E_{r}^{C}E_{+}^{C}-(1-E_{r}^{C})E_{-}^{C}\\ = & E_{r}^{C}(\alpha_{r}-1)(E_{+}^{C}-E_{-}^{C})+E_{r}^{C}\alpha_{r}\Delta \mu_{+}+(1-E_{r}^{C}\alpha_{r})\Delta \mu_{-}\\ = & \left(E_{+}^{C}-E_{-}^{C})\{E_{r}^{C}(\alpha_{r}-1)+\alpha_{r}E_{r}^{C}\frac{\Delta \mu_{+}}{E_{+}^{C}-E_{-}^{C}}+(1-\alpha_{r}E_{r}^{C})\frac{\Delta \mu_{-}}{E_{+}^{C}-E_{-}^{C}}\right\} \end{array}$$

$d_{+}$ and $d_{-}$ can be calculated as follows:

$$\begin{array}{cc} & d_{+}=\frac{\Delta \mu_{+}}{\left(E_{+}^{C}-E_{-}^{C}\right)}\\ & d_{-}=\frac{\Delta \mu_{-}}{\left(E_{+}^{C}-E_{-}^{C}\right)} \end{array}$$

where, $d_{+}$ and $d_{-}$ represent the normalized shift in the gene expression value corrected using the difference in the expected value of the gene expression value between the subsets.

As a result, the following Eq. 6 can be derived:

$$E[Y^{D}]-E[Y^{C}]=(E_{+}^{C}-E_{-}^{C})(E_{r}^{C}(\alpha_{r}-1)+\alpha_{r}E_{r}^{C}d_{+}+(1-\alpha_{r}E_{r}^{C})d_{-})$$

## Appendix B The detail of Eq. 8

The following properties of the variance and covariance properties of the two random variables A and B are used:

(B.1) V[A+B]=V[A]+V[B]+2Cov[A,B]

The covariance can be expressed using expected values, as follows:

(B.2) Cov[A,B]=E[AB]-E[A]E[B]

The following equation holds for the variance of the product of independent random variables.

(B.3) $$V[\mathrm{AB}]=V[A]V[B]+E{\left[A\right]}^{2}V[B]+E{\left[B\right]}^{2}V[A]$$

Using these formulas, the variance V[Y] of the bulk expression value can be expressed mathematically as follows, where we let $E_{+},E_{-},E_{r},V_{+},V_{-}$ and $V_{r}$ be equivalent to $E[\mu_{+}],E[\mu_{-}],E[r],V[\mu_{+}],V[\mu_{-}],V[r]$ respectively.

(B.4) $$\begin{array}{cc} V[Y]= & V[r\mu_{+}+(1-r)\mu_{-}+e]\\ = & V[r\mu_{+}+(1-r)\mu_{-}]+V[e]\\ = & V[r\mu_{+}]+V[(1-r)\mu_{-}]+2\mathrm{Cov}[r\mu_{+},(1-r)\mu_{-}]+V[e] \end{array}$$

$V[r\mu_{+}],V[(1-r)\mu_{-}]$ and $\mathrm{Cov}[r\mu_{+},(1-r)\mu_{-}]$ can be transformed into the following equations, respectively:

$$V[r\mu_{+}]=V_{r}V_{+}+E_{r}^{2}V_{+}+E_{+}^{2}V_{r}$$

$$\begin{array}{cc} V[(1-r)\mu_{-}]= & V[1-r]V_{-}+E{\left[1-r\right]}^{2}V_{-}+E_{-}^{2}V[1-r]\\ = & V_{r}V_{-}+{\left(1-E_{r}\right)}^{2}V_{-}+E_{-}^{2}V_{r} \end{array}$$

$$\begin{array}{cc} \mathrm{Cov}[r\mu_{+},(1-r)\mu_{-}]= & E[r(1-r)\mu_{+}\mu_{-}]-E[r\mu_{+}]E[(1-r)\mu_{-})]\\ = & E[r(1-r)]E_{+}E_{-}-E_{r}E_{+}E[1-r]E_{-}\\ = & (E_{r}-E[r^{2}])E_{+}E_{-}-E_{r}E_{+}(1-E_{r})E_{-}\\ = & E_{r}E_{+}E_{-}-E[r^{2}]E_{+}E_{-}-E_{r}E_{+}E_{-}+E_{r}^{2}E_{+}E_{-}\\ = & E_{+}E_{-}(E_{r}^{2}-E[r^{2}])\\ = & -E_{+}E_{-}V_{r} \end{array}$$

As a result, from Eq. B.4, Eq. 8 can be derived as follows:

$$\begin{array}{cc} V[Y]= & V[r\mu_{+}]+V[(1-r)\mu_{-}]+2\mathrm{Cov}[r\mu_{+},(1-r)\mu_{-}]+V[e]\\ = & V_{r}V_{+}+E_{r}^{2}V_{+}+E_{+}^{2}V_{r}+V_{r}V_{-}+{\left(1-E_{r}\right)}^{2}V_{-}+E_{-}^{2}V_{r}\\ & -2E_{+}E_{-}V_{r}+V[e]\\ = & {\left(E_{+}-E_{-}\right)}^{2}V_{r}+E_{r}^{2}V_{+}+{\left(1-E_{r}\right)}^{2}V_{-}+(V_{+}+V_{-})V_{r}+V[e] \end{array}$$
